# Older adult living independently in a public rowhouse project after the 2011 Fukushima earthquake: A case report

**DOI:** 10.1002/ccr3.5271

**Published:** 2022-01-11

**Authors:** Naomi Ito, Yuri Kinoshita, Tomohiro Morita, Sho Fujioka, Masaharu Tsubokura

**Affiliations:** ^1^ Division of Health and Welfare Soma City Government Soma Japan; ^2^ Department of Radiation Health Management School of Medicine Fukushima Medical University Fukushima Japan; ^3^ Division of Food Science and Nutrition Tohoku Seikatsu Bunka Junior College Miyagi Japan; ^4^ Department of Public Health School of Medicine Fukushima Medical University Fukushima Japan; ^5^ Department of Internal Medicine Soma Central Hospital Soma Japan

**Keywords:** aging‐in‐place, dementia, disaster, earthquake, Fukushima, rowhouse

## Abstract

We study an older Japanese woman who lived independently with minimal nursing or informal support from others in the rowhouse after the 2011 Fukushima disaster. This case report supports the effectiveness of *Idobata nagaya* as a measure of the municipality and offers an evidence‐based approach to reconstruction after a disaster. Considering the global population aging and isolation trends, the lesson from this case may apply to other settings beyond disasters.

## 1 INTRODUCTION

There is a universal desire in most people to continue living in familiar areas.^1^, ^2^ This is the central concept of community living, or “aging‐in‐place.”^3^ Aging‐in‐place helps older adults to maintain and improve their independence, dignity, and quality of life. However, aging‐in‐place is often affected by outside factors, such as disasters or diseases.^4^ Even though this is an important issue, adequate measures are often not taken in such emergencies. Planning sufficiently to realize aging‐in‐place in such cases is a critical issue for public health.^5^

When disaster strikes, achieving aging‐in‐place can be difficult for several reasons. Disasters affect the structure and function of families.^6^ For example, prolonged evacuation after a disaster forces younger generations to build new lives in evacuation destinations. Conversely, older generations tend to stay in the areas where they have lived for many years.^7^ Families, many of which are multi‐generational, are then separated due to the relocation of the younger generations, which leave aging parents alone at the old location.^8^

Concerns have recently arisen regarding the magnitude of emotional distress older adults experience when having to move to a new place and the support they would receive in their daily living that they had previously received from their extended family. Furthermore, due to the high percentage of older adults suffering from dementia, a growing interest has emerged nationally and internationally concerning ways to ensure patients' quality of life. In the event of a disaster, these measures are essential; however, knowledge on how to implement aging‐in‐place is still lacking.

After the 2011 triple disaster of earthquake, tsunami, and nuclear plant accident in Fukushima prefecture, many residents were forced to evacuate and relocate.^9^ Soma City, located in Hamadori in the eastern part of Fukushima prefecture, was one of the areas hit the hardest by the tsunami. The catastrophe caused many older people to be separated from their families. As a countermeasure, the Soma City government developed a rowhouse project as part of its post‐disaster recovery scheme.

While the government also offers detached houses that allows residents to live by themselves in a single unit, the rowhouse structure enables them to interact easily. Having already explored the ways to address the isolation issue among the older population, the government designed this rowhouse project in the hope that interactions among residents would create a culture of mutual support.

In this case report, we consider the situation of an 87‐year‐old woman with suspected mild dementia who moved to the rowhouse project after the earthquake and who continued to live independently with informal assistance. While many national governments build restoration housing projects in response to residential needs after natural disasters, the effects of this type of public housing project on the aging‐in‐place capabilities of older adults with medical conditions have not been widely investigated. This case report will therefore inform policy, practice, and research in the care of older individuals, both in times of emergency and normalcy.

## 2 CASE REPORT

The participant is an 87‐year‐old woman who currently lives in a rowhouse public project in Soma City, her hometown. After the 2011 earthquake, she evacuated her home, relocated to temporary housing twice, and in January 2014, she moved to the rowhouse. At the time, she had been diagnosed with hypertension and was taking medication for this condition. She was also suspected of having mild dementia; however, as she had never wanted to consult with a doctor for her possible dementia‐related symptoms, no diagnoses or treatments had ever been provided.

Her family lives some distance away—her son 50 km from her and her daughter 330 km—so they can only visit her monthly. Because of her medical needs, her family found it impossible for her to live in a house alone.

The housing project was in the form of a rowhouse and was called Soma *Idobata Nagaya*. Details of Soma *Idobata Nagaya* are first provided in the work of Yoshida et al.^10^ The term *Nagaya* originated from Japan's Edo‐style townhouse with individual rooms separated by thin walls, along with shared wells, restrooms, and alleys. *Idobata* refers to a space around the communal well where people chatted and offered to help one another.

The rowhouse community offers many services: supervision by a housing director and a manager, lunch delivery services, free shuttle bus services, a mobile grocery truck, and visits by volunteers and counselors. As this form of communal living consists of numerous interactions among residents and frequent visits among them, the client receives informal supervision and help from other residents.

The client was able to walk on her own with a walker cane and able to manage daily living slowly, with supervision. In 2016, after moving into the row house, the client's care team and fellow residents of the rowhouse noticed mild dementia symptoms in her behaviors. She repeated the same words or phrases. She missed medication occasionally and kept leftover medicine piled up in her room, resulting in worsened hypertension. She was taking fewer baths and often bought food from the grocery truck, not remembering what she had in stock.

She had piles of food in her room that had expired and her forgetful remarks and occasional public urinary incontinence led to complaints among residents. Even though she had a gentle personality, the client became a source of distress for the rowhouse's other residents. It was clear that she needed more support to compensate for her cognitive and physical impairments.

In January 2017, a physician advised her to apply to the government for nursing care services, and she began to receive level 2 care. Care level 2 is a level where you can live at home with the help and watching you need.^11^ Her family and care staff met regularly to discuss her needs. She preferred to receive help from a helper visiting at home, rather than going to a day‐care nursing facility for care. Her nursing care plan included a 1‐h cleaning and housekeeping service three times per week and bathing support. In December 2018, her benefits were increased to include homecare helper services six times a week and the cooking of daily meals. As a result, she now had a clean room as well as assistance for bathing and cooking.

She spent most of her time in the rowhouse, except for her monthly doctor's appointment, which she attended with her helper. She also attended community events—including tea parties and health consultation sessions—that took place several times a month; here, she enjoyed interactions with other residents. The client has a kind character, and the residents reported finding her excellent company.

The client's next‐door neighbor helps her with grocery shopping, mail collection, and adjusting her room temperature. They are close friends who share a morning ritual of reading newspapers together while having tea and an evening ritual of making sure that her blanket is tucked in appropriately. The client describes her neighbor as dependable and more than just a friend.

**FIGURE 1:**
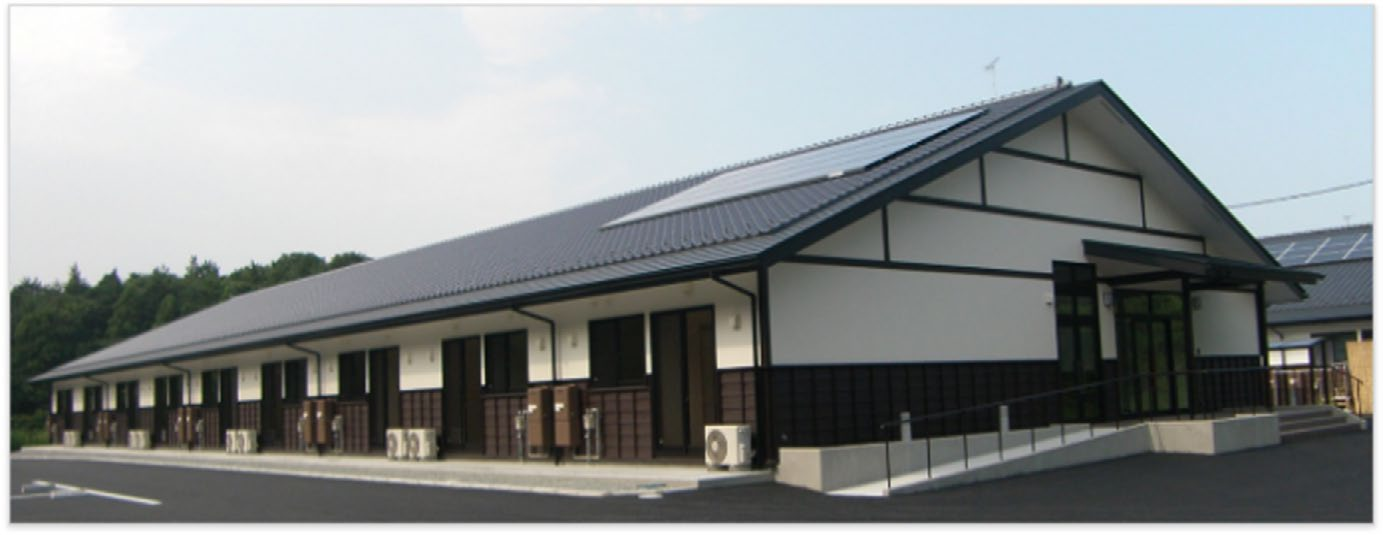
Front view of *Idobata Nagaya*. The building style is known as nagaya, a rowhouse commonly inhabited by ordinary people in downtown areas during the Edo period. Five buildings containing 58 units were built in Soma City after the earthquake

The client lives comfortably and happily, and her cognitive level and abilities remain stable. With daily help for an hour and monthly visits by her family, she lives an independent life in a rowhouse in her hometown. She has come to utilize a variety of support networks that have creatively emerged in the rowhouse since its inception. Had the disaster not taken place, she would have likely needed residential or institutional care. However, since she moved to the rowhouse, she has successfully maintained a good quality of life with informal support from her neighbors and part‐time formal support from government services (Figure 1).

## 3 DISCUSSION

This case study illustrates the possibility of an older individual with dementia living happily with non‐family members. By staying in a familiar community and reconnecting with its people, the older person can realize aging‐in‐place, in other words remaining in the town that they choose for as long as they want. The sense of connectedness to the place and its people allows for a life of independence and respect.

This case study also demonstrates the positive effects of the rowhouse setting on older adults with dementia. Stakeholders with diverse backgrounds and talents are connected through the operation of the rowhouse. Along with family, these stakeholders support the older individual, thereby providing informal care. With proper supervision, residents' fluid interactions promote a culture of mutual support and informal care.^12^ While some established care models for the elderly can be costly—such as the Continuing Care Retirement Community in the United States and elderly group homes for dementia care in Japan—the rowhouse facility is publicly funded and therefore more affordable. With supervised mutual support and informal care, older adults' need for nursing care services can be minimal, possibly reducing nursing care costs.

Moving to a different place in the later years can be emotionally challenging. In this case, moving turned out to be relatively easy because residents had no choice due to the disaster and subsequent loss of their houses. Further, the government developed a rowhouse project near the original location of the community. Therefore, people who moved to the rowhouse maintained and nurtured the local community's culture,^13^ an ideal recognized by the philosophy of comprehensive community care that promotes living in a familiar place.

While we acknowledge the uniqueness of this case's context, a city that was damaged by a disaster, its results are still useful and can be generalized to the broader context of the aging society in Japan and the rest of the world. With the challenges of population aging and the changing roles within the family, building communities with support networks is crucial for the well‐being of older adults. The rowhouse community's case is a great example of successfully ensuring quality of life for older adults.^10^ Future research should focus on older adults with ailments other than dementia within the rowhouse project to determine their outcomes in this setting.

## CONFLICT OF INTEREST

The authors declare that they have no competing interests.

## AUTHOR CONTRIBUTIONS

NI conceived the study and drafted the manuscript. YK, TM, SF, and MT conceived the study, participated in the study design and coordination, and helped draft the article. All authors have read and approved the final manuscript.

## CONSENT

This research meets the ethical guidelines and adheres to the local legal requirements of Japan. For this type of article, an ethical review was not required. Written informed consent was obtained from the patient.

## DATA AVAILABILITY STATEMENT

The data that support the findings of this study are available from the corresponding author upon reasonable request.
